# Acupoint herbal plaster for patients with primary dysmenorrhea: study protocol for a randomized controlled trial

**DOI:** 10.1186/s13063-018-2682-8

**Published:** 2018-07-03

**Authors:** Siyi Yu, Yueqiang Wen, Wanting Xia, Mingxiao Yang, Zhengtao Lv, Xiaoji Li, Wenyao Li, Sha Yang, Youping Hu, Fanrong Liang, Jie Yang

**Affiliations:** 1 The Department of Acupuncture and Tuina, Chengdu University of Traditional Chinese Medicine, Chengdu, Sichuan China; 2 The Department of Basic Medical Sciences, Chengdu University of Traditional Chinese Medicine, Chengdu, Sichuan China; 3 The Department of Clinical Medical, Chengdu University of Traditional Chinese Medicine, Chengdu, Sichuan China; 4 Department of Orthopedics, Tongji Hospital, Tongji Medical College, Huazhong University of Science and Technology, Wuhan, China

**Keywords:** Acupoint herbal plaster, Primary dysmenorrhea, Randomized controlled trial, Placebo control

## Abstract

**Background:**

Primary dysmenorrhea (PD), is one of main gynecological complaints in women of child-bearing age. Common medications for PD do not always achieve satisfactory outcome of pain relief. Hence, both health professionals and patients are seeking help from complementary and alternative medicine. The acupoint herbal plaster (AHP), which appears to be a safe and effective way to alleviate menstrual pain, as well as to improve other PD-related symptoms. Despite similar clinical studies for this condition in the past, no high-quality methodology-based clinical trial has been reported to date. The current study aims to assess the efficacy of the AHP compared with the acupoint placebo plaster (APP) and being placed on a waiting-list control group in patients with primary dysmenorrhea.

**Methods/design:**

This study is a randomized, single-center, placebo-controlled clinical trial. A total of 180 women with PD will be included and randomly allocated to the AHP, APP and waiting-list (WL) groups in a 1:1:1 ratio. Patients in the AHP group will be provided with herbal plasters (*Shaofuzhuyu* decoction) on various acupoints: *Shenque* (CV8), *Guanyuan* (CV4), *Qihai* (CV5), *Ciliao* (BL32) and *Zigong* (EX-CA1). Women in the APP group will receive placebo plasters on the same acupoints, and no intervention will be given to the WL group until completion of the study. The primary outcome will be pain intensity reduction measured by a Visual Analog Scale (VAS), with other outcome measurements including the Cox Menstrual Symptom Scale (CMSS), the 12-Item Short Form Health Survey (SF-12) and the Participant Global Impression of Change (PGIC). All assessments will be performed at baseline, each menstrual cycle during the treatment course and the follow-up course. Any adverse events will be recorded throughout the study.

**Discussion:**

This is the first study to compare the changes in menstrual pain after three different interventions: the active intervention (AHP), the placebo intervention (APP), and a period of no intervention (WL). This three-arm randomized controlled trial (RCT) aims to investigate the relative contributions of the specific (AHP vs. APP) and non-specific (APP vs. WL) effects to the overall clinical effects of the active AHP on women with PDM. The scientific and rigorous methodology design of this trial should gather good evidence to assess the curative effects and safety of the AHP on PD. Moreover, the results of this study may provide evidence-based references for the treatment of menstrual pain in future.

**Trial registration:**

Chinese Clinical Trial Registry, ID: ChiCTR-TRC-16008701. Registered on 22 July 2016.

**Electronic supplementary material:**

The online version of this article (10.1186/s13063-018-2682-8) contains supplementary material, which is available to authorized users.

## Background

Primary dysmenorrhea (PD) is prevalent among adolescent girls and women of reproductive age [1]. The incidence of PD ranges from 45% to 72% of all menstruating women; moreover, among adolescent girls it can be as high as 93% [2, 3]. Its symptoms vary but typically include dull, throbbing and cramping pain in the lower abdomen during menstruation [4, 5]. Patients may also experience vomiting, nausea, diarrhea, fatigue, fever, headache, sleeplessness and backache [6, 7]. This widespread condition not only decreases social participation and the quality of life of women, but also causes significant financial losses due to inability to work and extra medical costs [8, 9].

Approximately 67% of patients with PD choose anodyne drugs to temporarily relieve pain [10]. Nonsteroidal anti-inflammatory drugs (NSAIDs) are well advocated as the first-line medication for PD [11]. However, the pharmacological treatment has been reported to be associated with adverse effects including indigestion, headache, drowsiness; and in 20%–25 of patients, failure to relieve the pain was reported [12]. Consequently, efforts have been made to identify effective, low-risk interventions [13, 14].

Acupuncture and moxibustion therapy has been used as a non-pharmacological analgesic method in China for hundreds of years. Some clinical studies have demonstrated that acupuncture and moxibustion can be as effective as, or even superior to, pharmacological drugs in relieving menstrual pain [15–17]. In addition, a number of systematic reviews and meta-analyses have also shown that acupuncture leads to overt reduction in the pain of dysmenorrhea [18, 19]. As a typical external treatment in acupuncture, the acupoint herbal plaster (AHP) is a non-invasive, minimal-dose, transcutaneously absorbed, pharmacological treatment modality. Technically, its performance is based on the classical acupuncture and herbal medicinal theories. The treatment starts with placing processed Chinese herbal paste on a medical sticky plaster and then applying the plaster on certain acupoints. It is used for different treatment purposes in a much gentler format but can still achieve almost the same results of needling acupuncture. The AHP has been long known for its classical use of relieving respiratory conditions including pediatric recurrent pneumonia [20], stable chronic obstructive pulmonary disease [21], asthma [22] and rhinitis [23], as well as other conditions such as acute pancreatitis [24] and male infertility [25]. Nowadays, this traditional therapy has been innovated and developed to treat a much wider range of conditions including PD. Accordingly, modern clinical observational studies [26–28] have also indicated that the AHP has the potential to safely and effectively alleviate menstrual pain and improve PD-related symptoms.

According to the basic Traditional Chinese Medicine (TCM) theory, the major actions of the AHP are promoting blood circulation and moving energy (*Qi*) within the human body. The underlying mechanism of the favorable effect of the AHP possibly originates from the dual effects of acupoint stimulation and drug actions. Based on different herbal formulas, the AHP has been reported to exert different therapeutic effects, including regulating cellular immune function, reducing pulmonary surfactant proteins, and reducing the severity of inflammation [29–32]. Modern pharmaceutical studies have demonstrated that paeoniflorin, petroleum ether-soluble fraction, eleven gingerols and others are the key chemical compound constituents within the herbs in the AHP [33–36]. The herbs that will be used in the plaster come from an ancient formula named “*ShaoFu-ZhuYu Tang*” (SFZYT).. This has been recognized as a safe and effective remedy to treat PD since the Qing Dynasty [37, 38]. An earlier pharmacological study showed that SFZYT inhibits the constriction of the uterine muscles and causes a favorable anti-inflammatory action [39]. Moreover, another study that employed PD-modeled rats indicated that the estradiol, oxytocin, progesterone, endothelin, β-endorphin and PGF2α were restored to normal levels after intake of SFZYT [33]. These bioactive components within SFZYT can be absorbed transcutaneously from the herbal plaster. Hence, they can play a crucial role in regulating the whole body.

Unfortunately, there is a lack of high-quality clinical trials until now that have assessed the efficacy and safety of the AHP for PD. Therefore, we designed this clinical trial to fill this gap. To our knowledge, this study is the first RCT to date with a placebo design to test the efficacy and safety of the AHP on PD.

## Methods/design

### Study design

This proposed study is a single-centered, randomized, placebo-controlled trial which was devised following the Consolidated Standards of Reporting Trials (CONSORT) Statement recommendations [40] and the Standardized Protocol Items: Recommendations for Interventional Trials (SPIRIT) guidelines Additional file 1 [41]. Ethical approval has been obtained from the Institutional Review Board (IRB) of the Teaching Hospital of Chengdu University of Traditional Chinese Medicine (Approval No. 2016KL–003). We registered the study on the Chinese Clinical Trial Registry (Registration No. ChiCTR-IOR-16008701) in 2016.

**Fig. 1:**
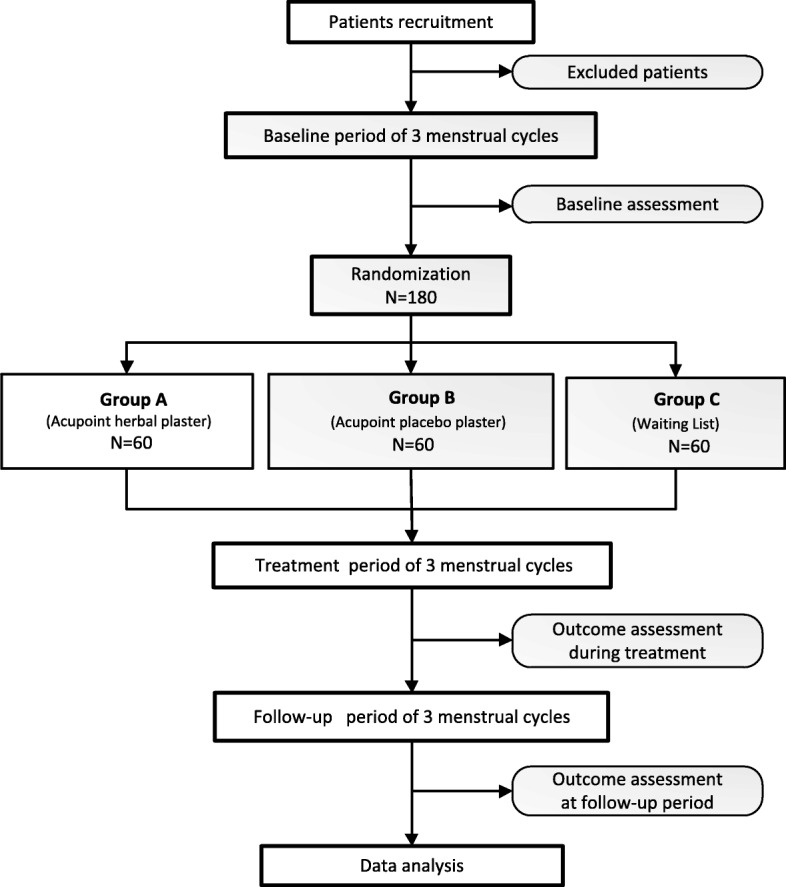
Flowchart of the trial

**Fig. 2:**
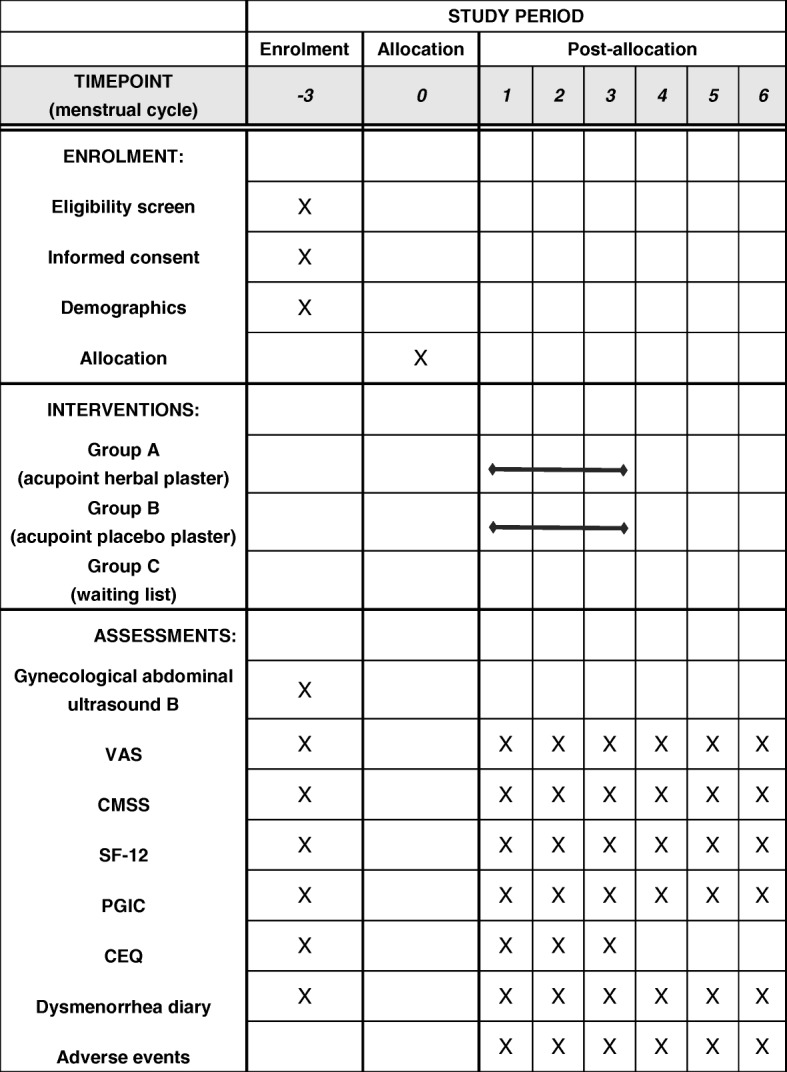
Standard Protocol Items: Recommendations for Interventional Trials (SPIRIT) Figure of enrollment, interventions and assessments. *VAS* Visual Analog Scale, *CMSS* Cox Menstrual Scale, *SF-12* 12-Item Short Form Health Survey, *PGIC* Participant Global Impression of Change, *CEQ* Credibility Expectancy Questionnaire

The study will take place in the 3rd Affiliated Hospital of Chengdu University of TCM. During a run-in period of three menstrual cycles prior to randomization, the patients will be required to complete a Dysmenorrhea Diary (DD) and record related symptom scores every month to establish an individualized baseline score. A total of 180 subjects meeting the including criteria will be allocated randomly to three research groups that include one experimental group of the AHP and two control groups of the acupoint placebo plaster (APP) group and the waiting-list WL group. After randomization, subjects in the AHP and APP groups will receive treatments for another three menstrual cycles. Follow-up assessments through telephone calls or social media smartphone application will be processed for the last period of three menstrual cycles until the completion of study. Altogether it takes 9 months of three stages with three period cycles in each session. (See Fig. 1 for the study flowchart, and Fig. 2 for the assessment schedule).

### Participants

#### Recruitment strategies

Study participants will be recruited mainly from the outpatient and inpatient sections of the Gynecology Department. Additionally, through flyers and social media advertisements, participants will be recruited from local communities and university campuses. All patients have the right to participate or drop out at any time, and will be required to sign the informed written consent before any study procedures commences.

#### Inclusion criteria

Participants who, in line with the following criteria, will qualify for the study are: (1) nulliparous women (18–30 years old), (2) women who meet the diagnostic criteria of PD under the Primary Dysmenorrhea Consensus Guideline [11], (3) women who meet the TCM diagnosis of “*qi* and blood stagnation” or “cold and dampness retention,” (4) women who have normal menstrual cycles (occurring every 28 ± 7 days), (5) a VAS score of the menstrual pain ≥ 40 mm during the baseline menstrual period, (6) women who agree to complete a DD during the study period, (7) women who agree to sign a informed consent form and volunteer for the research.

#### Exclusion criteria

Participants from the study who meet one or more following criteria will be excluded: (1) women with secondary dysmenorrhea caused by endometriosis, polycystic ovarian syndrome, pelvic inflammation and other gynecologic diseases, (2) women with any of the serious contraindications of cardiovascular, liver and kidney diseases, (3) women who are unable to express their subjective symptoms due to intellectual or mental conditions, (4) women with a malignant tumor, (5) women with poor compliance (i.e., cannot understand the purpose of this study or complete a DD during the baseline assessment), (6) pregnancy or breastfeeding, (7) women receiving another therapy or having received any other medical inventions for PD within the last 3 months.

### Randomization and allocation concealment

After the screening visit, qualified subjects will be randomly assigned to either the AHP group, the APP group, or the waiting-list group in a ratio of 1: 1: 1. Random numbers will be generated by a third person (BJ Gou) who is not practicing throughout the study, using the random number generator in the SPSS statistical software package (Version 22.0, SAS Institute Inc.). As the letters are drawn (A or B or C), they will be placed into opaque envelopes labeled with sequential numbers. The envelopes will be sealed and remain in numerical order in a safe place until the completion of surgery. The same researcher (BJ Guo) (not involved in the study) will prepare the envelopes. However, it was not possible to conceal the allocation among the waiting-list group.

### Blinding

The herbal paste will be packed and labeled by the 3rd Affiliated Hospital of Chengdu University of TCM to ensure that the practitioner and the patients involved in the study will remain fully blinded as to the identity of the treatment administered. Therefore, the participants, clinical practitioners, the outcome evaluators, the data manager and statistician will not know the treatment allocations, which will not be revealed until the end of study. In addition, the practitioner will be instructed not to communicate with participants about the possibility of their allocation. However, blinding of the waiting-list group is not possible for this trial.

### Interventions

**Fig. 3:**
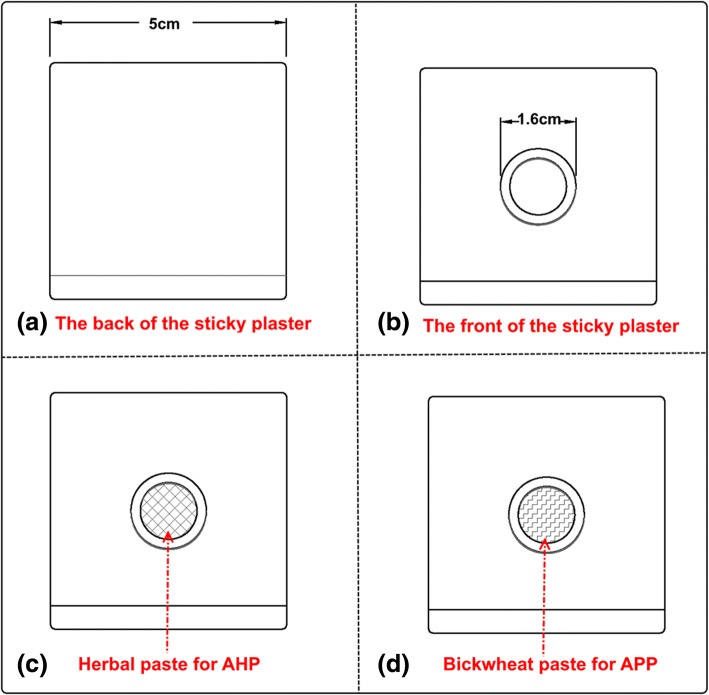
Illustration of the sticky plaster. **a** and **b** show the anterior and posterior appearance of the acupoint plaster, respectively. **c** and **d** show the paste inside the plaster. **c** demonstrates the herbal plaster. **d** demonstrates the bluckwheat plaster. These two kinds of paste are indentical in appearance. The Figs. are only for illustrative purposes

Acupoint plaster treatment will be performed by certified acupuncturists with at least 5 years’ clinical experience (Fig. 3a and b). All treatment details will be standardized between practitioners by guiding videos and relevant training before the first treatment session. The acupoint selection regimen was based on academic literature data mining [42] and expert opinions. Five acupoints will be used: *Shenque* (CV8), *Guanyuan* (CV4), *Qihai* (CV5), *Ciliao* (BL32) and *Zigong* (EX-CA1).

In this study, there are two types of paste to be applied on the sticky plasters: herbal paste (HP) for the AHP group, and buckwheat paste (BP) for the APP group (Fig. 3c and d). The HP remedy in this study comes from an ancient Chinese herbal formula named *Shaofu Zhuyu Tang* (SFZYT) which was recorded in a TCM medical classic, *Yi Lin Gai Cuo (Correction of Errors in Medical Classics)*, which was written or recorded by *Qingren Wang* in 1830. Clinical studies have shown the positive effect of SFZYT on PD [43, 44]. A systematic review recently found the therapeutic effects of SFZYT on PD to be superior to drugs [45]. In addition, previous clinical observations [46–48] have demonstrated that the SFZYT was safe for acupoint plaster. Therefore, we adapted this formula and standardized its performance.

In the AHP group, the standard operating procedures (SOP) are listed as follows:

1. Prepare the herbal materials: SFZYT includes *Angelicae sinensis Radix* (*danggui*), Rhizoma (*chuanxiong*), *Paeoniae Radix rubra* (*chishao*), *Cinnamomi cortex* (*rougui*), *Foeniculi fructus* (*xiaohuixiang*), *Zingiberis rhizoma (ganjiang*), Myrrha (*moyao*), *Trogopterus dung* (*wulingzhi*), Typhae Pollen (*puhuang*) and *Corydalis rhizoma* (*yanhusuo*). The ingredients of this formula are mixed in a weight ratio of 3: 1: 2: 1: 0.5: 2: 1: 3: 1: 1 and then the raw materials ground into fine powder (under 75 μm diameter) using a special Chinese medicinal crop powder mixer (manufactured by Royalstar Co., Ltd., Hefei, China)
2. Add the liquid base: place the mixed herbal powder into a container, add honey and stir well to make a thick herbal paste
3. Add the sticky plaster: put 10 g of the herbal paste onto a medical sticky plaster. Then apply the plaster over the acupoints. The sticky plasters are sized 5 × 5 cm^2^ with a 0.8-cm radius in the middle of which is placed the host herbal paste. The rest of the sticky plaster carries no herbal paste, which makes it adhere to the skin over the acupoints
4. Treatment procedures: locate the acupoints, disinfect the skin, and attach the plaster on the skin over the acupoints. The plasters will be left on the skin for 4–8 h depending on each patient’s individual response because it must be removed immediately should any allergic reaction occur, which may be described by patients as a burning sensation, severe itchiness, blisters, pruritus or rash on the local skin. A total of nine sessions will be performed during the three menstrual cycles, the treatment starts 1 week prior to the menstruation, with one 6–8-h treatment three times per week, according to the subject’s estimated dates

For the APP group, the placebo paste is made of buckwheat flour without any herbal medicines; however is still mixed with honey to maintain a similar appearance to the AHP (Fig. 2c and d) so that the plasters used in these two experimental groups are undistinguishable to patients and practitioners. Moreover, the only variable between the AHP group and the APP group is the ingredients of paste. In order to reduce the bias caused by performance, all the rest of the clinical procedures remain the same.

No intervention will be performed in the WL group. The participants are required to continue their current living habits, including exercise and diet, while filling out related questionnaires throughout the full observational period of six menstrual cycles. To meet the requirements of the Rule of Ethics, all participant in the WL group will be offered free AHP treatment of their choice at the completion of the study.

With regard to quality control of the herbal plaster, we will take the following measures: (1) first, the herbal plaster will be prepared beforehand by the central pharmacy of the 3rd Affiliated Hospital of Chengdu University of TCM that meets the requirements of the regulatory guidance issued by the China Food and Drug Administration, (2) in addition, pre-job SOP training and examination will be provided by the principal investigator (Prof. J Yang) to the practitioner for ensuring the quality of treatment procedures (i.e., locate the acupoints, disinfect the skin, attach the plaster on the skin over acupoints), (3) furthermore, the data and results will be monitored by the Evidence-based Medicine Center of Chengdu University of Traditional Chinese Medicine.

### Outcome measurements

#### Primary outcome

The primary outcome will be pain intensity reduction measured by the Visual Analog Scale (VAS). The severity of menstrual pain will be estimated using the VAS graded from 0 mm to 100 mm, indicating an absence of pain to the most serious pain imaginable [49].

#### Secondary outcomes

The secondary outcomes include the Cox Menstrual Symptom Scale (CMSS) [50], the 12-Item Short Form Health Survey (SF-12) [51], the Participant Global Impression of Change (PGIC) [52] and the DD.

#### Time points of outcome measurements

The VAS, CMSS, SF-12 and DD will be recorded at baseline and at every menstrual cycle throughout the treatment and the follow-up periods. The PGIC will be measured at the first, second, third, fourth, fifth and sixth menstrual cycles after inclusion.

We will estimate the expectation of participants to determine whether their expectations affected the outcomes by using the Credibility Expectancy Questionnaire (CEQ) [53, 54] at the baseline and the first to the third menstrual cycle before receiving treatment. In the proposed study, the questionnaire will be used to control for placebo effects, which are expected to be relatively high for light-therapy studies [54]. Blinding will be assessed by asking participants what group they thought they were in (AHP group or APP group, or “don’t know”) at the end of trial, and why they believed that they were in that group.

### Safety evaluation

Clinical adverse events (AEs) will be recorded throughout the study and graded using the National Cancer Institute Common Terminology Criteria for Adverse Events (CTCAE) version 4 [55]. According to previous randomized controlled trials (RCTs) [56, 57], the AHP may cause potential adverse events of itchiness, allergic responses of the skin or the whole body, local infections and other mild side effects. Any unexpected symptoms or signs during the treatment must be documented regardless of their relation to the study intervention. All details of related and unexpected AEs, such as time of occurrence, severity of AE, and suspected causes, will be recorded on Case Report Forms (CRFs). Participants with mild and moderate AEs will receive symptomatic treatment and will be closely followed up by the research team. Severe AEs will be reported to the Research Ethics Committee within 48 h.

### Sample size calculation

A few scientific trials have investigated the biomechanical effects of acupoint plaster on PD; however, the research methodologies are not appropriate to reference the determination of sample size to this study. The article being used for our study, was from an acupuncture clinical report with the use of VAS scores [16]. Liu’s study reported that patients who received classical acupoint achieved a mean reduction of 36.1 in VAS after acupuncture; while patients who received non-acupoint treatment showed a worse score of 29.9. Therefore, the decrease in VAS score of the three groups was predicted to be 38, 29 and 26, respectively. Using a bilateral test with an alpha value of 5% and a power of 80%, standard deviation for each group was 20 mm. According to the calculation with PASS software (Version 11.0, NCSS, LLC. Kaysville, UT, USA) in a 1:1:1 ratio, the effect size is 0.2550 of 51 participants in each group. The sample size is also calculated using the following formula [58]:

$$ n\kern0.5em =\kern0.5em 2{\left(\sigma \frac{z_{1-\alpha /\left(2\tau \right)}+{z}_{1-\beta }}{\mu_A-{\mu}_B}\right)}^2. $$

Allowing for a 15% dropout, the sample size was increased to 60 per group, making a total of 180.

### Statistical analysis plan

#### Data integrity

All participants are assessed at run-in period and at the first, second, third, fourth, fifth and sixth menstrual cycles after recruitment. A well-trained assessor, who is blinded to the treatment assignment, will collect clinical data using CRF files at each visit. Another two pre-trained data managers will verify and cross-check the CRFs, for the sake of ensuring reliability and accuracy of the data. If there are any queries in the CRFs, the results will be sent to the third investigator for resolution. Data managers will also be responsible for administration, coordination and monitoring (including Excel spread sheet-set up, data entry, coding and query management). Any incomplete data will be coded as unknown, missing or not applicable, and all data will be anonymously extracted to keep the identities of patients confidential. Results of the analysis must not be released with individual identification of the subject until the Excel spread sheet is closed.

#### Methods of statistical analyses

Data analyses will be processed with SPSS 22.0 (SPSS Inc., Chicago, IL, USA) and supervised by a skilled statistician blinded to group allocation.

The Full Analysis Set (FAS) is for all participants who have been randomized in terms of the intention-to-treat (ITT), and the per-protocol analysis set (PPS) and is for the individuals who complete the trial and do not have significant protocol violations. On the basis of rule of the last-observation-carried-forward, missing values will be imputed. In order to evaluate therapeutic effect and safety, the FAS and PPS will be used simultaneously.

Categorical data, such as gender and medical history, will be tabulated with frequencies or percentages; and continuous data, such as age and disease course, will be reported as mean ± standard deviation (SD), or median. For the baseline variables, sociodemographic data and other basic indicators will be carried out using analysis of variance (ANOVA) and *χ*^2^. To compare variables before and after treatment in the same group, a paired *t* test will be used. Repeated measures analysis of variance (ANOVA) will be used to compare the intergroup differences among the three groups. Tests of ITT between the AHP and APP arms and between the APP and WL arms with respect to the change will based on time-intervention interactions in the mixed-effect models. A *P* value of less than 0.05 (two-sided) indicates a statistically significant difference, with 95% confidence intervals.

## Discussion

Currently, there have been no methodologically controlled clinical studies confirming the efficacy of AHP therapy. To meet the demand for high-quality RCTs, our team has designed this randomized placebo-controlled trial to explore the potential therapeutic effect of the AHP for PD. In this pilot study, we will compare the change in menstrual pain after administration of three interventions: the active intervention (AHP), the placebo intervention (APP), and a period of no intervention (WL). This three-arm RCT aims to investigate the relative contributions of the specific (AHP vs. APP) and the non-specific (APP vs. WL) effects to the overall clinical effects of the AHP on women with PDM. In particular, a growing body of studies [58, 59] has confirmed that the sham control may play a crucial part in evaluating the effectiveness of a treatment, which may help separate the specific and non-specific effects. Therefore, the active and sham acupoint plasters should be adopted to investigate the specific analgesic effect on patients with PDM. In order to minimize the placebo effect, buckwheat flour paste is used in the placebo arm, which may successfully blind the patients and the practitioners due to their identical appearance. For the purpose of comparing the non-specific (sham intervention vs. no treatment) effects and avoiding bias caused by psychological influences, we set the third group of the waiting-list control without any acupuncture interventions. This is because participants in the APP group may have a placebo effect due to the unawareness of group allocation and the treatment that they are undergoing; therefore, placebo effects may happen secondary to their strong beliefs of being in the AHP group.

To our knowledge, the present study will be the first clinical trial that compares the AHP with the APP, and being placed in a waiting-list control group in treating PD. The scientific and rigorous methodologic design of this trial hopefully will provide consolidated evidence on the efficacy and safety of the AHP for treating PD, and hopefully provide reference to clinical practice Additional file 2.

### Trial status

Participant recruitment is currently ongoing.

## Additional files

Additional file 1: Standard Protocol Items: Recommendations for Interventional Trials (SPIRIT) 2013 Checklist: recommended items to address in a clinical trial protocol and related documents. (DOC 123 kb)

Additional file 2: Standards for Reporting Interventions in Clinical Trials of Acupuncture (STRICTA). (DOCX 17 kb)

## Abbreviations

AEs: Adverse events
AHP: Acupoint herbal plaster
ANOVA: Analysis of variance
AP: Acupoint plaster
APP: Acupoint placebo plaster
BP: Buckwheat plaster
CMSS: Cox Menstrual Symptom Scale
CONSORT: Consolidated Standards of Reporting Trials
CRF: Case Report Form
DD: Dysmenorrhea Diary
FAS: Full analysis set
HP: Herbal plaster
IRB: Institutional Review Board
ITT: Intention-to-treat
NSAIDs: Nonsteroidal anti-inflammatory drugs
PD: Primary dysmenorrhea
PGIC: Participant Global Impression of Change
PPS: Per-protocol analysis set
RCT: Randomized controlled trial
SD: Standard deviation
SF-12: 12-Item Short Form Health Survey
SFZYT: *Shaofu-Zhuyu Tang*
SOP: Standard operating procedures
TCM: Traditional Chinese Medicine
VAS: Visual Analog Scale
WL: Waiting list
