# Supplementary material for: Acupoint herbal plaster for patients with primary dysmenorrhea: study protocol for a randomized controlled trial
Source: Trials. 2018 Jul 3;19:348. doi: 10.1186/s13063-018-2682-8 (PMC6029355; doi:10.1186/s13063-018-2682-8)
Supplement: Supplementary file 2 — Standards for Reporting Interventions in Clinical Trials of Acupuncture (STRICTA). (DOCX 17 kb) [file 13063_2018_2682_MOESM2_ESM.docx]

**Checklist for items in STRICTA 2010**

| **Item** | **Detail** | **Page number** |
| --- | --- | --- |
| **1. Acupuncture rationale**            [(Explanations and examples)](http://www.stricta.info/explanations%20item%201.htm) | 1a) Style of acupuncture (e.g. Traditional Chinese Medicine, Japanese, Korean, Western medical, Five Element, ear acupuncture, etc) | 8-9 |
|  | 1b) Reasoning for treatment provided, based on historical context, literature sources, and/or consensus methods, with references where appropriate | 8-9 |
|  | 1c) Extent to which treatment was varied | 8-9 |
| **2. Details of needling**     [(Explanations and examples)](http://www.stricta.info/explanations_item_2.htm) | 2a) Number of needle insertions per subject per session (mean and range where relevant) | NA |
|  | 2b) Names (or location if no standard name) of points used (uni/bilateral) | 8 |
|  | 2c) Depth of insertion, based on a specified unit of measurement, or on a particular tissue level | NA |
|  | 2d) Response sought (e.g. *de qi* or muscle twitch response) | NA |
|  | 2e) Needle stimulation (e.g. manual, electrical) | NA |
|  | 2f) Needle retention time | 9 |
|  | 2g) Needle type (diameter, length, and manufacturer or material) | NA |
| **3. Treatment regimen**       [(Explanations and examples)](http://www.stricta.info/explanations_item_3.htm) | 3a) Number of treatment sessions | 6 |
|  | 3b) Frequency and duration of treatment sessions | 9 |
| **4. Other components of treatment**       [(Explanations and examples)](http://www.stricta.info/explanations_item_4.htm) | 4a) Details of other interventions administered to the acupuncture group (e.g. moxibustion, cupping, herbs, exercises, lifestyle advice) | 8-9 |
|  | 4b) Setting and context of treatment, including instructions to practitioners, and information and explanations to patients | 8 |
| **5. Practitioner background**     [(Explanations and examples)](http://www.stricta.info/explanations_item_5.htm) | 5) Description of participating acupuncturists (qualification or professional affiliation, years in acupuncture practice, other relevant experience) | 8 |
| **6. Control or comparator interventions**     [(Explanations and examples)](http://www.stricta.info/explanations_item_6.htm) | 6a) Rationale for the control or comparator in the context of the research question, with sources that justify this choice | 9,12 |
|  | 6b) Precise description of the control or comparator. If sham acupuncture or any other type of acupuncture-like control is used, provide details as for Items 1 to 3 above. | 8-9 |

Note: This checklist, which should be read in conjunction with the explanations of the STRICTA items, is designed to replace [CONSORT 2010’s item 5](http://www.consort-statement.org/consort-statement/3-12---methods/item5_interventions/) when reporting an acupuncture trial.
